# Lower Internal Additive Noise and Better Perceptual Template Characterize Binocular Contrast Sensitivity Summation

**DOI:** 10.3389/fpsyg.2021.740759

**Published:** 2021-09-30

**Authors:** Pan Zhang, Hanlin Wang, Weicong Ren, Qing Lu, Chenxi Li, Ge Chen, Shilei Zhang, Jiayu Tao, Ying Li, Di Wu, Zeng Wang

**Affiliations:** ^1^Department of Psychology, Hebei Normal University, Shijiazhuang, China; ^2^Library, Hebei Medical University, Shijiazhuang, China; ^3^School of Nursing, Yueyang Vocational Technical College, Yueyang, China; ^4^College of Art and Design, Zhengzhou University of Light Industry, Zhengzhou, China; ^5^Huihua College, Hebei Normal University, Shijiazhuang, China; ^6^Department of Psychology, Chengde Medical University, Chengde, China; ^7^Department of Psychiatry, Beijing Children’s Hospital, Capital Medical University, National Center for Children Healthy, Beijing, China; ^8^Military Medical Psychology School, Air Force Medical University, Xi’an, China; ^9^Department of Psychology, Hebei Medical University, Shijiazhuang, China

**Keywords:** contrast sensitivity, binocular summation, internal additive noise, perceptual template, spatial frequency

## Abstract

Binocular summation is generally defined as the superiority of binocular over monocular performance. Here, we investigated how external noise modulates the effect of binocular summation on the contrast sensitivity function (CSF) and clarified the corresponding mechanisms with a perceptual template model (PTM). The contrast sensitivity (CS) over 10 spatial frequencies and three external noise levels was assessed under one binocular and two monocular viewing conditions. The binocular summation ratio (BSR) was calculated by dividing the area under the log CSF (AULCSF), or the CS of using both eyes, by that of only using the “good eye” (BSRG) or the “bad eye” (BSRB), respectively. We found that: (1) based on the AULCSF, the BSRB was higher than the BSRG; (2) based on the AULCSF, the BSR was more pronounced under zero-noise than under low-noise conditions, but the BSR was not higher than 1 under high-noise conditions due to a large individual difference; (3) based on the CS, with increasing spatial frequencies, the BSRB steadily increased; (4) both decreased internal additive noise and an improved perceptual template accounted for the gain in binocular summation. These results help us better understand the features of binocular CS and shed light on the clinical studies on populations with monocular CS loss.

## Introduction

The phenomenon that visual performance when using both eyes is better than using a single eye is called binocular gain or binocular summation. This advantage of the binocular viewing condition may be explained by the probability summation and/or neural summation of the signals from the two eyes (Campbell and Green, 1965; Blake and Fox, 1973). For example, both binocular summation and inhibition in the striate cortex have been confirmed by single-cell recording experiments (Crawford and Cool, 1970; Li and Creutzfeldt, 1984; Ohzawa and Freeman, 1986).

As a fundamental feature of visual function, contrast sensitivity (CS) reflects the ability to detect luminance differences between adjacent areas (Campbell, 1983; Pelli and Bex, 2013). Contrast sensitivity provides useful information, which may not be obtained from a traditional visual acuity test (Rubin et al., 1997). Thus, this study mainly focused on binocular CS summation. It has been demonstrated that the extent of binocular CS gain depends on many factors. For example, Home found that subjects produce greater binocular gain when processing low contrast stimuli than when processing high contrast stimuli (Home, 1978). Targets at high spatial frequencies result in greater binocular gain than those at low spatial frequencies (Pardhan, 1996), and increasing eccentricity impairs the binocular gain (Pardhan, 1997; Alberti and Bex, 2018). In addition, older adults often exhibit a low efficiency of binocular summation compared with young adults (Pardhan, 1997).

Previous studies have found that the monocular CS difference can strongly modulate binocular summation (Pardhan and Gilchrist, 1990a,b). Specifically, when the CS in two eyes is equal, maximum binocular gain occurs. In contrast, the binocular gain gradually becomes weak as the monocular CS difference increases. Binocular CS is lower than monocular CS after monocular CS difference beyond a critical degree, which is called binocular inhibition, and this phenomenon has been confirmed in clinical populations with amblyopia and patients with unilateral cataract (Pardhan and Elliott, 1991).

This study investigated whether the summation of binocular CS is modulated by spatial frequency and external noise. The contrast sensitivity function (CSF) is a curve that denotes the relationship between CS and spatial frequency, and it characterizes the fundamentals of spatial vision. Furthermore, the CSF has great applications in both basic and clinical research studies. However, a traditional CSF assessment requires a huge number of trials, which limits its applications. Fortunately, within a Bayesian framework, scientists have created the quick CSF (qCSF) algorithm, which could precisely and accurately estimate the whole CSF with fewer trials (Lesmes et al., 2010). The qCSF algorithm has been further validated in many studies (Hou et al., 2010, 2015; Zhang et al., 2018; Xi et al., 2020; Yan et al., 2020). In this study, the qCSF method was used to investigate the binocular CS gain over extensive spatial frequencies within a short time.

Little is known about how binocular gain varies in noisy environments. In real life, it is quite common to detect a target in a noisy background, e.g., identifying a pedestrian during foggy weather. On one hand, adding various levels of external noise helps researchers better understand the features of the CSF. On the other hand, with the external noise method, a perceptual template model (PTM) can clarify the intrinsic limitations of human perception by measuring the CS at different external noise levels (Lu and Dosher, 2008). The PTM decomposes the limitations of perception into the following three independent factors: (1) internal additive noise, which is equal to amplifying both signal and noise from input stimuli; (2) perceptual template, which changes the ability of external noise exclusion; (3) internal multiplicative noise, which follows Weber’s law (Dosher and Lu, 1998). The PTM has been successfully used to explain how luminance changes visual function (Li et al., 2015) and the improvements induced by reward and perceptual learning (Dosher and Lu, 1998; Zhang et al., 2018). As such, the combination of the external noise method and the PTM is an ideal tool for understanding the features and mechanisms of binocular summation.

Therefore, this study has two aims: (1) to examine whether and how the binocular summation of CS is modulated by spatial frequency and external noise; (2) to determine the corresponding mechanisms with the PTM.

## Materials and Methods

### Participants

Nine participants (18–23 years of age) with normal or corrected-to-normal vision were recruited. Before the experiment, the participants signed consent forms. The Ethical Review Committee of Hebei Normal University approved the procedure of this study, which also followed the Declaration of Helsinki.

### Laboratory Apparatus

A luminance-calibrated cathode-ray tube (CRT) monitor (Dell monitor, resolution 1280 × 1024; 85 Hz) was used to display the stimuli. The monitor was controlled by MATLAB with the Psychophysics Toolbox (Pelli, 1997). The subjects put their heads on a chin rest and sat 171 cm from the screen comfortably. The background luminance was 34.7 cd/m^2^.

### Stimuli

The targets were vertical gratings that were displayed in the central visual field. The gratings were at 10 spatial frequencies (0.5, 0.67, 1, 1.33, 2, 2.67, 4, 5.33, 8, and 16 cpd) and three external noise levels [μ = 0 and σ∈ (0, 0.12, and 0.24)]. The cycle was constant (*N* = 3). Thus, the spatial frequencies of the gratings were inversely proportional to their sizes. To blur the edge, each grating was covered with a truncated Gaussian envelope. The sizes of the noise images and gratings (before the envelope) were identical. The number of noise elements in each image was set as a fixed number (15 × 15) so the noise images could produce the same spectrum energy as the gratings under every spatial frequency condition (Chen et al., 2014).

### Procedure

**FIGURE 1:**
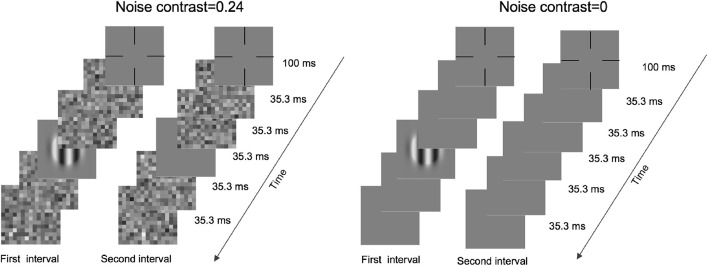
Illustration of a typical trial under high- (left) and zero- (right) noise conditions.

An illustration of a typical trial is shown in Figure 1. Each trial was initialized by a fixation cross and lasted 100 ms. Then, two intervals, divided by a 500-ms blank, were presented in the center of the monitor. Under zero-noise conditions, each interval included two frames of blanks, one frame of blank or grating, and another two frames of blanks. Under noise conditions, the two front and back blanks were replaced by noise images. Each frame lasted 35.3 ms, and each noise image was randomly sampled from the same noise distribution. In addition, these images differed across trials, intervals, and frames. The subjects were asked to report which interval included grating by pressing the buttons of a game controller. A brief beep was presented after each response regardless of correctness.

### Design

The whole CSF test consisted of three noise levels (0, 0.12, and 0.24) and 10 spatial frequency conditions, which were measured by the qCSF procedure (Hou et al., 2010). The qCSF procedure was developed based on two critical assumptions as follows: (1) an individual CSF can be well fitted by a model with four parameters; (2) the slopes of psychometric functions do not depend on spatial frequency or external noise intensity (Rohaly and Owsley, 1993; Strasburger, 2001; Watson and Ahumada, 2005; Chen et al., 2014). The three noise levels were randomly mixed between trials. Each noise condition included 100 trials. The test sequences of two monocular conditions and one binocular condition were counterbalanced between subjects. For monocular testing, the non-tested eye was covered with an opaque occluder. To better evaluate the binocular gain, we computed the area under the log CSF (AULCSF, in log_10_ units), which served as the index of CS across all spatial frequencies (Koop et al., 1996; Zhang et al., 2018; Wu et al., 2020, 2021). For each subject, eyes with larger and smaller AULCSF were defined as good and bad eyes, respectively. The binocular summation ratio (BSR) was computed by the performance (AULCSF or CS) in the binocular condition divided by that of good (BSRG) or bad (BSRB) eyes.

### Perceptual Template Model Model Analysis

In the PTM, the performance of a subject was calculated using the following equation:


(1) d=′(β⁢c)γ(Af⁢Ne⁢x⁢t)2⁢γ+Am2⁢Nm⁢u⁢l2⁢((β⁢c)2⁢r+(Af⁢Ne⁢x⁢t)2⁢γ)+(Aa⁢Na⁢d⁢d)2

where *d*′ denotes the performance; the equivalent internal additive and multiplicative noise is expressed by *N*_*add*_ and *N*_*mul*_, respectively; *N*_*ext*_ indicates the contrast of external noise; γ denotes the non-linearity of the system; β is the perceptual template gain; *c* presents the signal contrast. To model the effect of binocular summation on CS, *A*_*a*_, *A*_*f*_, and *A*_*m*_ were added in front of *N*_*add*_, *N*_*ext*_, and *N*_*mul*_, respectively. Under monocular conditions, *A*_*a*_, *A*_*f*_, and *A*_*m*_ were all set to 1. Because the slope was found to be constant between the binocular and any one of the two monocular conditions (see detailed analysis in Supplementary Information), the multiplicative noise was assumed to be constant and independent of the viewing condition. Thus, we removed *A*_*m*_ from Equation 1 and only set *A*_*a*_ and *A*_*f*_ free (Xu et al., 2006; Zhang et al., 2018). In addition, under different spatial frequency conditions, *N*_*a*_ and β varied, but *N*_*m*_ and γ did not. In summary, two potential factors were considered to explain the binocular gain on CS: lower internal additive noise and a better perceptual template (the ability of the external noise filter). The full model assumed that binocular summation decreased internal additive noise and improved the perceptual template. The reduced model 1 assumed that binocular summation only decreased internal additive noise, and the reduced model 2 assumed that binocular summation only improved the perceptual template. The most reduced model assumed no changes in the parameters. The goodness of fit was calculated as follows:


(2) $$r^{2}=1-\frac{\sum {\left(y_{i}-{\hat{y}}_{i}\right)}^{2}}{\sum {\left(y_{i}-\bar{y}\right)}^{2}}$$

where *r*^2^ is the index of the goodness of fit; $\,{\hat{y}}_{i}$ and *y*_*i *_denote the predicted and original values, respectively; $\bar{y}\,$ represents the mean of all original values. To select the best model, we performed an *F*-test to compare the *r*^2^ of the four models (Huang et al., 2010). The best-fitting model was the one that was statistically better than any reduced model but not significantly worse than the full model.

## Results

**FIGURE 2:**
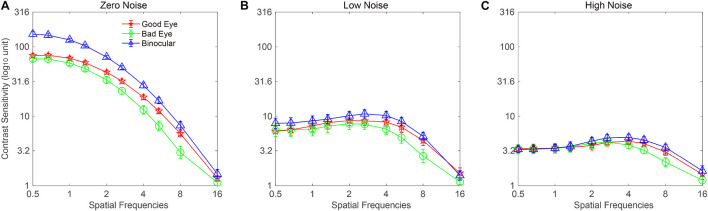
Monocular and binocular contrast sensitivity functions (CSFs) at **(A)** zero-, **(B)** low-, and **(C)** high-noise levels, respectively. Red lines with pentagram symbols, green lines with circle symbols, and blue lines with triangles denote contrast sensitivity (CS) when using the good eye (GE), bad eye (BE), and two eyes (TE), respectively. Data were averaged across subjects. The error bar denotes SE.

**FIGURE 3:**
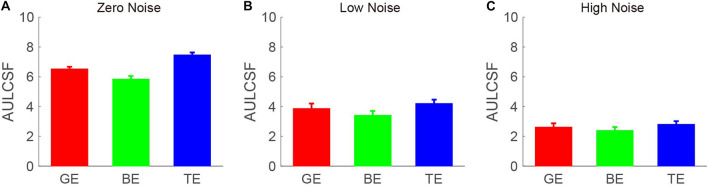
Area under the log CSF (AULCSF) (log_10_ units) under **(A)** zero-, **(B)** low-, and **(C)** high- **(C)** noise conditions. Red, green, and blue bars denote the data from GE, BE, and TE, respectively. The error bar denotes SE.

The CSF at the three noise levels when using the good eye, the bad eye, and both eyes are plotted in Figure 2. A visual inspection suggested that the binocular CS was much better than any monocular CS, especially when external noise was absent. To briefly compare the CS between monocular and binocular conditions, the AULCSF is plotted (Figure 3). The AULCSF of the good eye (GE), bad eye (BE), and two eyes (TE) for the conditions was as follows: 6.527 ± 0.147, 5.842 ± 0.211, and 7.478 ± 0.15 (log_10_ unit, mean ± SE) under zero-noise conditions, respectively; 3.872 ± 0.328, 3.414 ± 0.288, and 4.212 ± 0.252 under low-noise conditions, respectively; 2.621 ± 0.254, 2.402 ± 0.226, and 2.815 ± 0.204 under high-noise conditions, respectively. A repeated measure ANOVA was conducted on the AULCSF with the eye and noise conditions as within-subject factors. The main effects of the eye and noise conditions and the interaction effect among them were all significant [*F*(2, 16) = 3.693, *p* < 0.001; *F*(2, 16) = 251.135, *p* < 0.001; *F*(4, 32) = 98.259, *p* < 0.001]. A simple-effect analysis revealed that: (1) when no noise was present, the AULCSF was largest in TE, followed by GE and BE (all, *p* < 0.05); (2) when low noise was present, the AULCSF was also largest in TE, followed by GE and in BE (all, *p* < 0.05); (3) when high noise was present, only the difference in the AULCSF between TE and BE reached marginal significance (*p* = 0.078). In summary, significant binocular CS dominance was observed, especially when zero or low noise was present.

**FIGURE 4:**
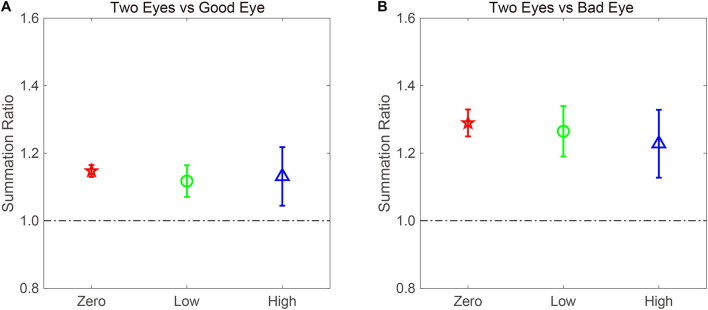
AULCSF-based binocular summation ratios (BSRs) when the **(A)** good and **(B)** bad eyes were considered as the standard eyes, respectively. Red, green, and blue colors denote the zero-, low-, and high-noise conditions, respectively. The error bar denotes SE.

The BSR was computed by dividing the binocular AULCSF by the AULCSF of the “good eye” (BSRG) or “bad eye” (BSRB), respectively (Figure 4). The BSRG under zero-, low-, and high-noise conditions was 1.147 ± 0.018, 1.118 ± 0.047, and 1.131 ± 0.087, respectively. In contrast, the BSRB under zero-, low-, and high-noise conditions was 1.29 ± 0.040, 1.265 ± 0.075, and 1.228 ± 0.101, respectively. On average, the BSRB was greater than the BSRG across different noise conditions (1.261 ± 0.052 vs. 1.132 ± 0.045). To provide statistical results, a repeated ANOVA was performed on the summation ratios with the standard eye (good vs. bad) and noise conditions (zero, low, and high) as within-subject factors. The main effect of the standard eye conditions was significant, indicating that the summation ratio was significantly higher when the bad eye was considered as the standard eye [*F*(1, 8) = 6.485, *p* < 0.001]. However, the main effect of noise conditions and the interaction effect between standard eye and noise conditions failed to reach significance [*F*(2, 16) = 0.175, *p* = 0.841; *F*(2, 16) = 0.192, *p* = 0.827].

Traditionally, a BSR with a value greater than 1 denotes binocular gain. However, if the BSR is smaller than 1, binocular loss or inhibition is indicated. Thus, it is quite necessary to confirm whether summation ratios were significantly different from 1. When the good eye was considered as the standard eye, a repeated analysis was conducted on the summation ratios to verify the difference between the three noise conditions and 1. We found that the main effect was marginally significant [*F*(3, 24) = 2.45, *p* = 0.088]. Least significant difference (LSD) test revealed that the summation ratios under zero- and low-noise conditions were significantly greater than 1 (all, *p* < 0.05), but that there was no difference between high-noise conditions and 1 (*p* = 0.169). When the bad eye was considered as the standard eye, the same analysis was conducted, and the results were similar. That is, the summation ratios under zero- and low-noise conditions were significantly greater than 1 (all, *p* < 0.05), but the difference between high-noise conditions and 1 reached marginal significance (*p* = 0.053). These findings suggested that external noise modulates the extent of binocular summation.

**FIGURE 5:**
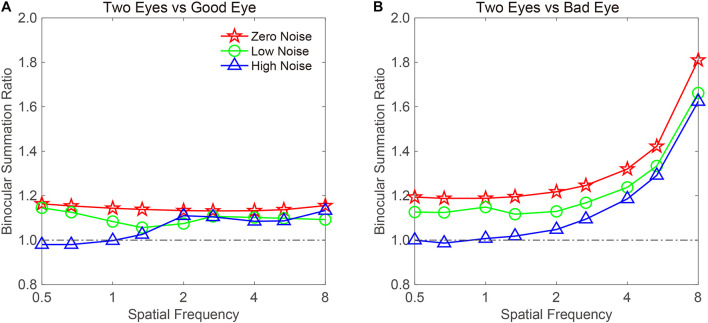
CS-based BSRs vs. a spatial frequency curve. Data were plotted when the **(A)** good or **(B)** bad eye was considered as the standard eye, respectively. Red, green, and blue colors denote the zero-, low-, and high-noise conditions, respectively.

To examine whether the binocular summation effect is dependent on spatial frequency, we plotted the CS-based BSR vs. a spatial frequency curve (Figure 5). When calculating the summation ratio, 16 cpd may produce some extreme values, because CS could not be measured at a specific external noise level on some subjects. To exclude the influence of extreme values on the data analysis, the data at 16 cpd were not included. For the remaining data, we averaged the CS in different noise conditions and across subjects. The BSR when the good eye was considered as the standard eye is plotted in Figure 5A. A visual inspection suggested that the BSR under zero- and low-noise conditions was greater than 1 at all spatial frequencies. On average, the BSRs were 1.143 ± 0.003 and 1.098 ± 0.009 across spatial frequencies under zero- and low-noise conditions, respectively. In contrast, the BSR under high-noise conditions was only 1.056 ± 0.02 across spatial frequencies. However, the pattern in the high-noise condition suggested that binocular summation gain only existed at high spatial frequencies. Indeed, the BSRs were 0.996 ± 0.011 and 1.104 ± 0.009, at low (<2 cpd) and high (≥2 cpd) spatial frequencies, respectively.

When the bad eye was considered as the standard eye, the BSR vs. the spatial frequency curve was plotted (Figure 5B). The BSR curve started horizontally at low spatial frequencies and then increased with spatial frequency. Under zero-noise conditions, the BSRs were 1.191 ± 0.002 and 1.403 ± 0.108 at low (<2 cpd) and high (≥2 cpd) spatial frequencies, respectively. Under low-noise conditions, the BSRs were 1.129 ± 0.007 and 1.306 ± 0.096 at low (<2 cpd) and high (≥2 cpd) spatial frequencies, respectively. Under high-noise conditions, the BSRs were 1.003 ± 0.007 and 1.249 ± 0.103 at low (<2 cpd) and high (≥2 cpd) spatial frequencies, respectively. These findings indicated that the BSR is highly dependent on spatial frequency, but high noise decreases it.

**FIGURE 6:**
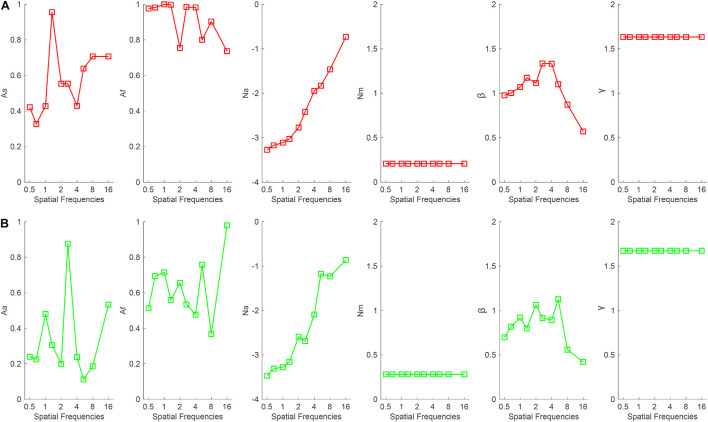
*A*_*a*_, *A*_*f*_, *N*_*a*_, *N*_*m*_, β, and γ as a function of spatial frequencies when the **(A)** good or **(B)** bad eye was considered as the standard eye, respectively.

To illustrate the mechanisms of binocular summation, the data were first averaged across subjects and then fitted with the PTM. The model with the least parameters while maintaining the significant effect of the binocular summation according to Equation 2 was selected as the best-fitting model. When the good eye was considered as the standard eye, the *r*^2^ of the full, reduced Model 1, reduced Model 2, and most reduced model was 96.3, 95, 87.2, and 86.8%, respectively. The full model was selected as the best-fitting model because it *r*^2^ was significantly higher than that of the reduced models (all, *p* < 0.05). On average, the *A*_*a*_ and *A*_*f*_ in the full model were 0.572 ± 0.059 and 0.912 ± 0.034, respectively, across all spatial frequencies (Figure 6A). When the bad eye was considered as the standard eye, the *r*^2^of the full, reduced Model 1, reduced Model 2, and most reduced model was 95.4, 78, 76.3, and 71.2%, respectively. The full model was selected as the best-fitting model because it *r*^2^ was significantly higher than that of the reduced models (all, *p* < 0.05). On average, the *A*_*a*_ and *A*_*f*_ in the full model were 0.34 ± 0.073 and 0.625 ± 0.055, respectively, across all spatial frequencies (Figure 6B). These results suggested that both decreased internal additive noise and the improved perceptual template contribute to the binocular summation.

## Discussion

In this study, we explored the effect of external noise and spatial frequency on binocular CS summation and determined the corresponding mechanisms with a PTM. The binocular gain was observed, but its extent was strongly dependent on the standard eye, spatial frequency, and external noise conditions. The PTM analysis suggested that the internal additive noise was reduced and that the perceptual template was promoted under binocular viewing conditions.

The spatial frequency-dependent binocular summation gain has been investigated by previous studies, but our experimental design still provided significant innovation. First, the CSF was accessed through the qCSF method with high precision and efficiency (Lesmes et al., 2010). Approximately 500–1,000 trials are required by the traditional CSF measurement to produce a reasonable precision, which is time-consuming (Harvey, 1986). Thus, the qCSF method not only saves working time but also reduces fatigue in subjects. Second, due to the limitation of the traditional CSF assessment, some researchers only measured two spatial frequencies (Pardhan, 1996). In contrast, the CS over 10 spatial frequencies was obtained with the qCSF method. Thus, a clear pattern of the BSR vs. the spatial frequency curve was observed, and the binocular summation gain was evaluated by both CS and AULCSF.

The external noise level-dependent BSR was another highlight of this study. First, based on the AULCSF, the BSR was greatest under zero-noise conditions, followed by low-noise and high-noise conditions. In addition, with high external noise, there was a greater individual difference in the BSR. Second, based on the CS, the BSRB increased with spatial frequencies regardless of external noise levels. In contrast, the BSRG was dependent on a spatial frequency only when high external noise was present.

Binocular summation ratios have been investigated by many studies (Frisén and Lindblom, 1988; Pardhan, 1996, 1997; Thylefors and Havelius, 2014). In most of these studies, the BSR is between 0 and 100%, largely depending on which visual task is studied. The BSRs in this study were lower than those determined with the contrast detection task. For example, when considering the good eye as the standard eye, the BSR in our study was 1.19 and 1.2 at 1 and 5.3 cpd, respectively. In contrast, Pardhan (1996) reported that the BSR was 1.45 and 2.26 at 1 and 5.3 cpd, respectively. This difference may be due to different experimental settings, e.g., stimuli size, duration time, and screen luminance. Pardhan and Shahia did not report the details of their duration time. The duration time in the current study was 33.3 ms, which was short. Thus, it will be quite interesting to examine the effect of duration time on the BSR in the future.

With the help of the PTM, we found that lower internal noise and a better perceptual template characterized the binocular summation gain. In addition, when the bad eye was considered as the standard eye, the changes in internal noise and perceptual template were much more pronounced than those when the good eye was considered the standard eye. These results were expected because the extent of the binocular gain was much greater when the bad eye was considered as the standard eye. Interestingly, the extent of the changes in internal noise was greater than that in the perceptual template, implying that the changes in internal noise are the dominant mechanism.

In this study, the grating size was varied with spatial frequencies. Although there may be a concern that CS is attributed to grating size instead of spatial frequency, there is evidence to suggest otherwise. First, Pelli and Bex suggested that the use of a fixed number of sinewave cycles is much better because neurons in the visual cortex are roughly spatial scale-invariant (Pelli and Bex, 2013). Second, when fixing the grating size, increasing spatial frequency may induce additional processes, e.g., spatial summation (Jamar and Koenderink, 1983). Third, the stimuli with fixed cycles have been used in many previous studies (Xu et al., 2006; Chen et al., 2014; Hou et al., 2015; Li et al., 2015; Zheng et al., 2018, 2019; Yan et al., 2020). Furthermore, fixing the cycles of grating maintains the spectral relationship between the signal and external noise remained identical under all the spatial frequency conditions. Thus, these studies suggest that the present experimental setting is rigorous.

The improvement of the binocular summation gain is an interesting research topic. A previous study has found that patients with large interocular CS differences, such as amblyopia (Pardhan and Whitaker, 2000), produced a low efficiency of binocular summation. The improvement in the visual function of the amblyopic eye by perceptual learning (Huang et al., 2008; Xie and Yu, 2018) or transcranial direct current stimulation (Ding et al., 2016) has been demonstrated. However, it is still unknown whether or how much the efficiency of binocular summation could be restored. The qCSF method with multiple external noise levels has great implications for the recovery of patients with ocular conditions, especially when they participate in a visual task in an extreme environment, e.g., external noise.

## Data Availability Statement

The original contributions presented in the study are included in the article/Supplementary Material, further inquiries can be directed to the corresponding author/s.

## Ethics Statement

The studies involving human participants were reviewed and approved by the Ethical Review Committee of Hebei Normal University. The patients/participants provided their written informed consent to participate in this study.

## Author Contributions

PZ, DW, and ZW designed the experiment and wrote the manuscript. PZ collected and analyzed the data. PZ, DW, HW, WR, QL, GC, CL, JT, SZ, and YL revised the manuscript. All authors contributed to the article and approved the submitted version.

## Conflict of Interest

The authors declare that the research was conducted in the absence of any commercial or financial relationships that could be construed as a potential conflict of interest.

## Publisher’s Note

All claims expressed in this article are solely those of the authors and do not necessarily represent those of their affiliated organizations, or those of the publisher, the editors and the reviewers. Any product that may be evaluated in this article, or claim that may be made by its manufacturer, is not guaranteed or endorsed by the publisher.
