# Supplementary material for: Lower Internal Additive Noise and Better Perceptual Template Characterize Binocular Contrast Sensitivity Summation
Source: Front Psychol. 2021 Sep 30;12:740759. doi: 10.3389/fpsyg.2021.740759 (PMC8514620; doi:10.3389/fpsyg.2021.740759)
Supplement: Supplementary file 1 [file Table_1.DOCX]

**Supplementary Information**

**Slope Check in Measurement of the Contrast Sensitivity Function**

We compared the slopes of the psychometric function between three viewing conditions.

The performance of an observer was computed by the following equation:

$P_{i,j}\left( x \right)=\gamma+\left( 1-\gamma-\lambda\right)\left( 1-exp\left( -{10}^{s\left( {log}_{10}\left( x \right)-{log}_{10}\left( \tau_{i,j} \right) \right)} \right) \right)$, Eq. S1

where *P_i,j_*(*x*) is percent correct in the *i*^th^ spatial frequency and *j*^th^ external noise level; guessing rate (*γ*) is 0.5 and lapse rate (*λ*) is 0.02; *τ* is the contrast threshold at 80.3% correct performance level; the unique free parameter is *s*, which denotes the slope of psychometric function.

The best fitted parameters were obtained by a maximum-likelihood procedure. A repeated measurement analysis was performed on the slopes with eye conditions (good eye, bad eye and binocular) as a within-subject variable. The main effect of eye condition was not significant (F(2, 16) = 0.489, *p* = 0.622), indicating that the slope was constant.

**Fitting the PTM**

The raw CSF data could derived one psychometric functions with six contrast level, corresponding to 60%, 70%, 78%, 84%, 90%, and 99% correct. Ten spatial frequencies, three external noise levels, and two eye conditions produced 60 psychometric functions. Thus, a total of 360 data points were included in model fitting.
